# Variations in the management of diffuse low-grade gliomas—A Scandinavian multicenter study

**DOI:** 10.1093/nop/npab054

**Published:** 2021-09-04

**Authors:** Bodil Karoline Ravn Munkvold, Ole Solheim, Jiri Bartek, Alba Corell, Eddie de Dios, Sasha Gulati, Eirik Helseth, Klas Holmgren, Margret Jensdottir, Mina Lundborg, Eduardo Erasmo Mendoza Mireles, Ruby Mahesparan, Øystein Vesterli Tveiten, Peter Milos, Henrietta Nittby Redebrandt, Lars Kjelsberg Pedersen, Jon Ramm-Pettersen, Rickard L Sjöberg, Björn Sjögren, Kristin Sjåvik, Anja Smits, Gregor Tomasevic, Tomás Gómez Vecchio, Einar O Vik-Mo, Maria Zetterling, Øyvind Salvesen, Asgeir S Jakola

**Affiliations:** 1 Department of Neuromedicine and Movement Science, Faculty of Medicine and Health Sciences, Norwegian University of Science and Technology, NTNU, Trondheim, Norway; 2 Department of Neurosurgery, St. Olavs Hospital, Trondheim University Hospital, Trondheim, Norway; 3 Department of Neurosurgery, Karolinska University Hospital, Stockholm, Sweden; 4 Department of Clinical Neuroscience, Karolinska Institutet, Stockholm, Sweden; 5 Department of Neurosurgery, Copenhagen University Hospital Rigshospitalet, Copenhagen, Denmark; 6 Institute of Neuroscience and Physiology, Sahlgrenska Academy, Gothenburg, Sweden; 7 Department of Neurosurgery, Sahlgrenska University Hospital, Gothenburg, Sweden; 8 Department of Neuroscience, Uppsala University, Uppsala, Sweden; 9 Department of Neurosurgery, Oslo University Hospital, Oslo, Norway; 10 Institute of Clinical Medicine, Faculty of Medicine, University of Oslo, Oslo, Norway; 11 Department of Clinical Sciences, Neuroscience, Umeå University, Umeå, Sweden; 12 Department of Neurosurgery, University Hospital of Northern Sweden, Umeå, Sweden; 13 Department of Clinical Medicine, Faculty of Medicine, University of Bergen, Bergen, Norway; 14 Department of Neurosurgery, Haukeland University Hospital, Bergen, Norway; 15 Department of Neurosurgery, Linköping University Hospital, Sweden; 16 Department of Clinical Sciences, Lund University, Lund, Sweden; 17 Department of Neurosurgery, Skåne University Hospital, Lund, Sweden; 18 Department of Neurosurgery, University Hospital of North Norway, Tromsø, Norway; 19 Department of Neurosurgery, Uppsala University Hospital, Uppsala, Sweden; 20 Department of Public Health and Nursing, Faculty of Medicine and Health Sciences, Norwegian University of Science and Technology, Trondheim, Norway

**Keywords:** adjuvant, chemotherapy, diagnostic imaging, glioma, radiotherapy, surgical oncology

## Abstract

**Background:**

Early extensive surgery is a cornerstone in treatment of diffuse low-grade gliomas (DLGGs), and an additional survival benefit has been demonstrated from early radiochemotherapy in selected “high-risk” patients. Still, there are a number of controversies related to DLGG management. The objective of this multicenter population-based cohort study was to explore potential variations in diagnostic work-up and treatment between treating centers in 2 Scandinavian countries with similar public health care systems.

**Methods:**

Patients screened for inclusion underwent primary surgery of a histopathologically verified diffuse WHO grade II glioma in the time period 2012 through 2017. Clinical and radiological data were collected from medical records and locally conducted research projects, whereupon differences between countries and inter-hospital variations were explored.

**Results:**

A total of 642 patients were included (male:female ratio 1:4), and annual age-standardized incidence rates were 0.9 and 0.8 per 100 000 in Norway and Sweden, respectively. Considerable inter-hospital variations were observed in preoperative work-up, tumor diagnostics, surgical strategies, techniques for intraoperative guidance, as well as choice and timing of adjuvant therapy.

**Conclusions:**

Despite geographical population-based case selection, similar health care organizations, and existing guidelines, there were considerable variations in DLGG management. While some can be attributed to differences in clinical implementation of current scientific knowledge, some of the observed inter-hospital variations reflect controversies related to diagnostics and treatment. Quantification of these disparities renders possible identification of treatment patterns associated with better or worse outcomes and may thus represent a step toward more uniform evidence-based care.

Diffuse low-grade gliomas (DLGGs, ie, WHO grade II) account for approximately 13% of all diffuse gliomas with an annual incidence rate of 1 per 100 000 person-years and typically affect a relatively young patient group, with median age of about 40-45 years at diagnosis.^1–3^ Although mild symptom burden and slow radiological growth often characterize early stage of disease,^4^ lesion expansion and malignant transformation into rapidly progressing high-grade gliomas (HGGs, ie, WHO grade III-IV) almost inevitably occur at some point, causing severe morbidity and dramatically deteriorated prognosis.^5–7^

Early extensive surgery of radiologically defined tumor prolongs time with tumor control and overall survival (OS).^1,8^ Moreover, improved OS has been demonstrated in “high-risk” patients treated with adjuvant radiation therapy (RT) and procarbazine, lomustine, and vincristine (PCV) compared to RT alone.^9–11^ Nevertheless, there are still controversies in DLGG management, related to diagnostic work-up, surgical strategies including technical aids for pre- and intraoperative guidance, and choice and timing of adjuvant treatment regimens.

A prerequisite for standardizing and optimizing treatment protocols is to obtain an overview over current standards at different centers that could motivate future comparative studies or trials. In the present retrospective multicenter population-based cohort study in a single-payer universal health care setting, we sought to explore possible variations in diagnostics and treatment strategies of DLGG across centers in 2 Scandinavian countries.

## Materials and Methods

### Study Design and Included Patients

The study is part of a collaborative Scandinavian multicenter project including all 11 neurosurgical departments performing glioma surgery in Norway and Sweden. Included centers appear from author affiliations. The Scandinavian tax-funded universal coverage public health care system, with geographical-based referral to regional neurosurgical centers, limits the possibility for referral bias. Since there are no competing private alternatives, insurance policies do not influence the management, and the study population thus represents a practically unselected population-based series. Regional tumor board meetings and discussion in multidisciplinary teams are endeavored in both countries, and national standardized clinical pathways for diagnostics, treatment, and follow-up on suspicion of a brain tumor were developed and implemented during the study period. All departments aim for tissue diagnostics upon radiological suspicion of a DLGG.

Patients screened for inclusion were adults 18 years or older who underwent primary surgery (biopsy or resection) of a histopathologically verified supratentorial diffuse WHO grade II glioma in the time period from 2012 through 2017. Tumors were classified according to the 2007 or 2016 WHO classification system.^12,13^ Three centers did not register data in 2017. Incidence rates and temporal trends were therefore calculated for the time period 2012-2016.

### Data Collection and Study Variables

Clinical and radiological data were retrieved from medical records at the respective institutions or collected as part of research projects conducted locally. Data were collected between August 2018 and September 2019, and study variables were filled out in electronic case report forms (CRF). The CRF covered patient characteristics, preoperative work-up, tumor data, and a detailed description of primary surgical care and adjuvant treatment regimens, as well as surgical approach at disease progression and recurrence. Dates of radiological diagnosis, primary surgery, and re-operation were also registered. An overview of the collected variables can be found in Supplementary Table S1. The study is reported in accordance with the STROBE (Strengthening the Reporting of Observational Studies in Epidemiology) guidelines.^14^

### Statistics

Statistical analyses were performed in IBM SPSS Statistics version 27.0 (IBM Corp., Armonk, NY, USA) and R version 3.6.3. All tests were 2-sided and statistical significance level was set at *P* ≤ .05. Holm’s sequential Bonferroni procedure was conducted to counteract family-wise error rate associated with multiple testing, and all reported *P* values are adjusted. Normality was explored using the Shapiro-Wilk test and by visual assessment of Q-Q plots, while distributional shapes were assessed by visual inspection of boxplots. Central tendencies are presented as mean (±SD) or median (range) for normally distributed and skewed data, respectively. Chi-square test, Fisher’s exact test, and Fisher-Freeman-Halton exact test were conducted for hypothesis testing in contingency tables. For r × c contingency tables, omnibus testing was supplemented with analyses of adjusted standardized residuals. Mann-Whitney *U* test and Kruskal-Wallis test were conducted for exploration of differences between groups on a continuous or ordinal dependent variable. Cochran-Armitage test of linear trends in proportions was conducted for investigation of temporal trends.

Population estimates were obtained from Statistics Norway and Statistics Sweden. Annual age-standardized incidence rates were calculated by adjusting the crude incidence rates relative to the European standard population 2011-2030 projection, as recommended in the Eurostat revision guidelines from 2013.^15^

Funnel plots were generated for exploration of inter-hospital variations in important key aspects of clinical management plotted against case volume. Unadjusted funnel plots were generated with observed proportion at each center plotted against case volume, with the observed overall proportion set as the target outcome for Y. The funnel control limits for identifying potential outliers were obtained at 95% and 99% prediction limits based on the Binomial distribution of proportions. Further, multivariable logistic regression modeling was used to risk-adjust outcome variations between centers based on preselected clinical factors. Resection rates within 6 months from radiological diagnosis were adjusted for patient age, Karnofsky performance status (KPS) score, and year of surgery. Rates of early postoperative RT plus PCV were adjusted for age, KPS score, year of surgery, and primary surgical intervention (initial biopsy only vs primary resection). Predicted probabilities of the event for each patient treated at each center were calculated and summarized to render expected count at each center. Adjusted ratio at each center was calculated by dividing the observed number of cases by the predicted number of cases at each center, ie, the observed-to-expected ratio. For the adjusted funnel plots, control limits for identifying potential outliers were obtained at 95% and 99% prediction limits based on the Poisson distribution.

### Ethics and Approvals

Data collection and transfer of patient data across treatment centers were approved by the Regional Committee for Medical and Health Research Ethics in Central Norway (REC reference 2017/1780) and by the regional committee of Western Sweden (EPN reference 705/17). The need for informed consent was waived by the committees.

## Results

### Incidence Rates and Patient Characteristics

Patient characteristics including symptom burden at radiological diagnosis and an overview of the presurgical work-up with range between treating centers are presented in Table 1, whereas temporal trends are displayed in Table 2. A total of 642 patients underwent primary surgery of a histopathologically verified DLGG in the study period, with case volumes ranging between 19 and 110 across the included centers. Crude annual incidence rates in the adult population per 100 000 were 1.1 in Norway and 0.9 in Sweden; age-standardized incidence rates were 0.9 and 0.8 in the 2 countries. The overall annual age-standardized incidence rate remained fairly stable throughout the 5-year period, with only a slight decrease from 0.8 to 0.7 from 2012 to 2016. Male:female ratio was 1:4 and did not differ by country, year of surgery, or histopathological subtype.

**Table 1.** Patient Characteristics, Symptom Burden at Radiological Diagnosis, Diagnostic Radiological Assessment, and Other Preoperative Work-up

	Total	Norway	Sweden
Number of patients, total (cases per center)	642 (19-110)	254 (19-80)	388 (36-110)
Age at surgery in years, overall median (lowest and highest median age across centers)	43 (36-51)	42 (36-46)	44 (41-51)
	Total, N (%, range %)^a^	Norway, n (%, range %)^a^	Sweden, n (%, range %)^a^
Female	264 (41, 35-60)	100 (39, 35-60)	164 (42, 36-48)
Symptoms at (radiological) diagnosis			
Seizure	382 (60, 44-76)	137 (54, 44-67)	245 (63, 46-76)
Cognitive deficit	86 (13, 0-25)	38 (15, 0-25)	48 (12, 8-16)
Motor deficit	91 (14, 0-22)	34 (13, 0-22)	57 (15, 8-22)
Language deficit	61 (10, 0-15)	25 (10, 0-15)	36 (9, 4-14)
Visual deficit	47 (7, 0-21)	18 (7, 0-21)	29 (7, 4-14)
Headache/ICP-related symptoms	152 (24, 15-38)	78 (31, 21-38)	74 (19, 15-25)
Asymptomatic/incidental	71 (11, 4-23)	33 (13, 7-23)	38 (10, 4-15)
Preoperative KPS score^b^			
80-100	555 (86, 64-100)	210 (83, 64-100)	345 (89, 72-96)
70	64 (10, 0-32)	35 (14, 0-32)	29 (7, 0-19)
<70	23 (4, 0-8)	9 (4, 0-4)	14 (4, 0-8)
Clinical deterioration preoperatively	36 (6, 0-15)	22 (9, 0-15)	14 (4, 1-6)
Diagnostic radiological work-up			
Structural MRI			
Contrast enhancement	203 (32, 20-39)	78 (31, 20-36)	125 (32, 25-39)
Multifocal	64 (10, 0-19)	34 (13, 0-19)	30 (8, 2-14)
Eloquence	378 (59, 41-72)	127 (50, 41-63)	251 (65, 54-72)
Missing	2 (0, 0-3)	0 (0)	2 (1, 0-3)
Largest tumor diameter prior to surgery in millimeters, overall median (lowest and highest median)	45, 40-60	40, 40-45	45, 40-60
Demonstrated tumor growth prior to surgery	107 (17, 0-22)	43 (17, 0-20)	64 (16, 9-22)
MR spectroscopy	244 (38, 8-98)	181 (71, 37-98)	63 (16, 8-29)
Positron emission tomography (PET)	37 (6, 0-38)	6 (2, 0-4)	31 (8, 0-38)
Preoperative functional brain mapping^c^			
Functional MRI (fMRI)	132 (35, 7-67)	56 (44, 20-67)	76 (30, 7-65)
Diffusion tensor imaging (DTI)	201 (53, 3-94)	80 (63, 18-94)	121 (48, 3-86)
Transcranial magnetic stimulation (nTMS)	79 (21, 0-55)	5 (4, 0-28)	74 (29, 0-55)
Other preoperative work-up			
Neuropsychological assessment	64 (10, 0-42)	13 (5, 0-19)	51 (13, 0-42)
If yes: Neuropsychological impairment	28 (44, 0-100)	2 (15, 0-100)	26 (51, 33-56)
Missing	4 (6, 0-100)	3 (23, 0-100)	1 (2, 0-100)

Abbreviations: ICP, intracranial pressure; KPS, Karnofsky performance status; MRI, magnetic resonance imaging.

^a^Total number N (%, with range between centers in %).

^b^Karnofsky and Burchenal.^16^

^c^Within surgical cases located in presumed eloquent brain regions as evaluated from structural MRI.

**Table 2.** Temporal Trends

	2012	2013	2014	2015	2016	2017^a^	Total^b^, N (%)
Number of cases	114	129	117	101	98	61	642
Age-standardized incidence rate	0.8	0.9	0.8	0.7	0.7	—	0.8
Norway	1.0	1.2	0.9	0.7	0.6	—	0.9
Sweden	0.7	0.8	0.8	0.7	0.7	—	0.8
Preoperative work-up							
MR spectroscopy	36 (32)	49 (38)	40 (34)	31 (31)	39 (40)	36 (59)	244 (38)
Amino acid PET	9 (8)	6 (5)	5 (4)	7 (7)	8 (8)	1 (2)	37 (6)
Preoperative DTI and/or fMRI^c^	33 (55)	41 (57)	39 (56)	33 (55)	44 (76)	33 (73)	231 (61)
Neuropsychological assessment	3 (3)	6 (5)	8 (7)	12 (12)	16 (16)	17 (28)	64 (10)
Initial surgical strategies							
Watch-and-scan	17 (15)	25 (19)	22 (19)	14 (14)	16 (16)	8 (13)	109 (17)
Resection within 3 months^d^	63 (55)	77 (60)	65 (56)	53 (52)	48 (49)	33 (54)	347 (54)
Resection within 6 months^e^	77 (68)	86 (67)	77 (66)	71 (70)	62 (63)	40 (66)	422 (66)
Intraoperative techniques							
Mapping or awake surgery^f^	15 (35)	25 (45)	32 (67)	26 (63)	31 (74)	24 (71)	157 (58)
Diagnosis							
Astrocytoma	49 (43)	68 (53)	56 (48)	59 (58)	52 (53)	35 (57)	330 (51)
Oligodendroglioma	32 (28)	39 (30)	39 (33)	27 (27)	44 (45)	26 (43)	215 (33)
Oligoastrocytoma	33 (29)	22 (17)	22 (19)	15 (15)	2 (2)	0 (0)	97 (15)
Molecular classification^g^	39 (34)	37 (29)	42 (36)	48 (48)	67 (68)	53 (87)	297 (46)
Adjuvant therapy							
Early RT + CHT	28 (25)	28 (22)	26 (22)	19 (19)	24 (24)	25 (41)	154 (24)
Early RT + PCV	5 (4)	4 (3)	10 (9)	10 (10)	13 (13)	10 (16)	54 (8)
Surgical resection within 6 months, followed by early RT + CHT	15 (13)	20 (16)	19 (16)	11 (11)	15 (15)	17 (28)	97 (15)
Surgical resection within 6 months, followed by early RT + PCV	2 (2)	2 (2)	7 (6)	3 (3)	7 (7)	8 (13)	29 (5)

Abbreviations: CHT, chemotherapy; DLGG, diffuse low-grade glioma; DTI, diffusion tensor imaging; fMRI, functional magnetic resonance imaging; IDH, isocitrate dehydrogenase; PCV, procarbazine, lomustine, and vincristine; PET, positron emission tomography; RT, radiotherapy; TMZ, temozolomide.

^a^Three centers did not register patients in 2017, and incidence rates were therefore not calculated for 2017.

^b^Year of primary surgery was missing in 22 cases, and the sum of cases may therefore not add up to 100% of the total case volume in the study period.

^c^Within cases located in presumed eloquent brain regions as evaluated from preoperative structural MRI.

^d^Surgical resection within 3 months from when a DLGG was radiologically suspected.

^e^Surgical resection within 6 months from when a DLGG was radiologically suspected.

^f^Mapping or awake surgery performed during primary resections in presumed eloquent locations (n = 270).

^g^Mutational status of both *IDH* and 1p/19q assessed.

### Clinical Presentation and Preoperative Work-Up

**Figure 1.**
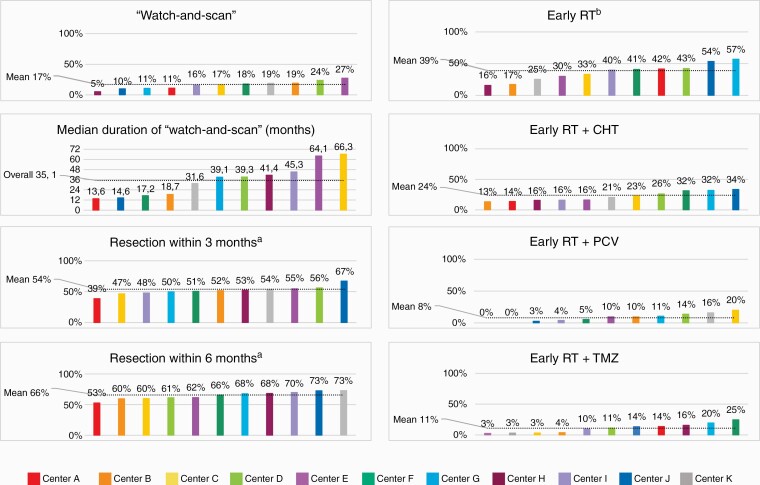

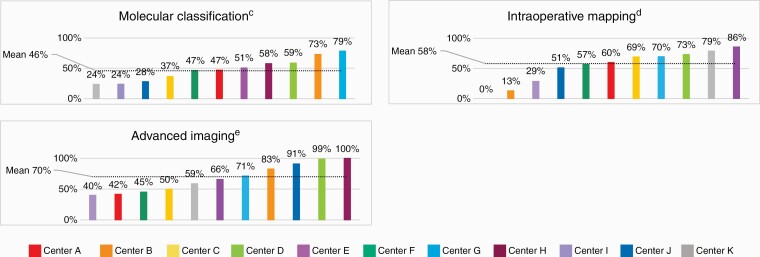
Variations in aspects of clinical management of diffuse low-grade glioma across the 11 included centers (A–K), ranked from lowest to highest percentage. The dotted line marks the mean percentage. CHT: chemotherapy; PCV: procarbazine, lomustine, vincristine; RT: radiotherapy; TMZ: temozolomide. ^a^Time from radiological diagnosis to surgical resection. ^b^Initiation of radiotherapy within 6 months postoperatively. ^c^Mutational status of *IDH* and 1p/19q assessed. ^d^Use of intraoperative brain mapping during tumor resections in presumed eloquent brain regions. ^e^Diffusion tensor imaging (DTI), functional magnetic resonance imaging (fMRI), magnetic resonance spectroscopy (MRS), positron emission tomography (PET).

**Figure 2.**
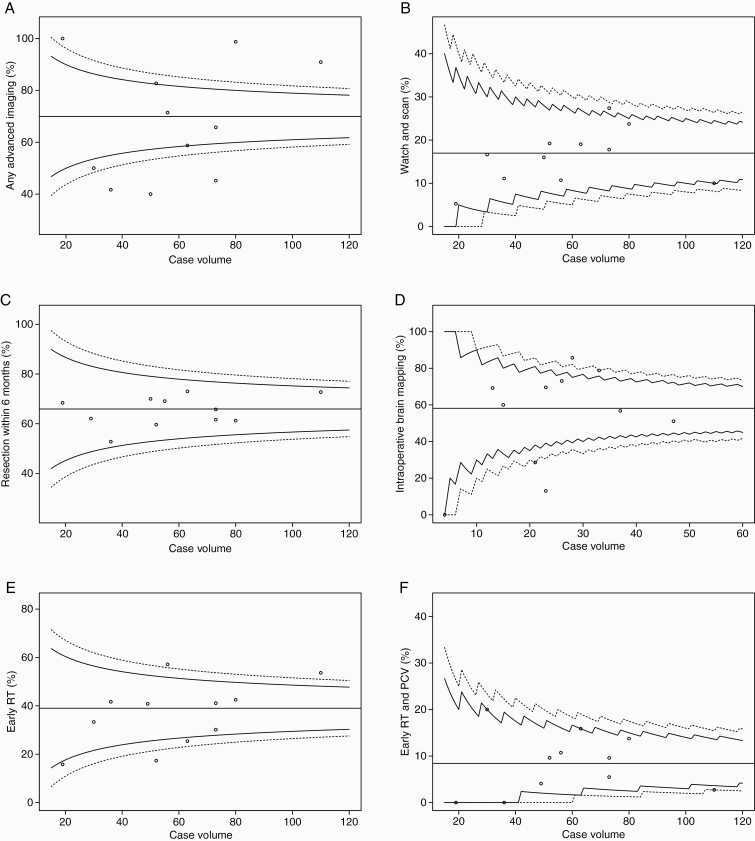
Funnel plots displaying rates of (A) advanced preoperative imaging (diffusion tensor imaging [DTI], functional MRI [fMRI], MR spectroscopy [MRS], positron emission tomography [PET]), (B) watch-and-scan, (C) resection rates within 6 months from radiological diagnosis, (D) intraoperative brain mapping during primary resections in presumed eloquent locations, (E) early radiotherapy, and (F) early postoperative radiotherapy plus PCV (procarbazine, lomustine, vincristine) plotted against case volume at each of the 11 treating centers. The target outcome for Y (horizontal solid line) is the observed overall proportion in percentage. The solid funnels represent 95% control limits, while the dotted funnels represent 99% control limits.

Epileptic seizures were the most frequent onset symptom (60%), followed by headaches and/or other symptoms related to increased intracranial pressure (ICP) (24%). Seventy-one tumors (11%) were incidentally discovered when neuroimaging was carried out for unrelated symptoms or disease. An overview of different aspects of clinical management at the individual treating centers is displayed in Figure 1. There were pronounced variations between the centers in the degree to which structural MRI was supplemented with advanced imaging techniques for more precise preoperative tissue diagnostics and functional brain mapping (Table 1, Figure 1). Any advanced imaging (ie, diffusion tensor imaging [DTI], functional magnetic resonance imaging [fMRI], magnetic resonance spectroscopy [MRS], and/or positron emission tomography [PET]) was carried out in 449 patients (70%), with diverging practices between treating centers (range 40%-100%). As displayed in the funnel plot in Figure 2, 7 centers are outside the 95% control limits, and there is no clear relation between case volume and the use of advanced imaging. Neuropsychological assessment was not part of standard preoperative work-up at any of the centers, but the prevalence increased by more than a 5-fold between 2012 (3%, 0%-25%) and 2016 (16%, 0%-54%) (*P* < .02).

### Tumor Classification

As seen in Figure 1, the implementation of molecular markers in tumor diagnostics varied much between the centers. Thirty-nine cases (25%) were classified according to the 2007 WHO Classification system after 2016, and only 2 tumors were classified as oligoastrocytomas after 2016. Patients harboring *IDH-mutant* tumors were significantly younger than patients diagnosed with *IDH-wildtype* tumors, with median age of 41 (18-77) years vs 54 (18-80) years at the time of primary surgery (*P* < .02). Further, 68% of centrally located tumors were *IDH-wildtype*, while only 8% were *IDH-mutant* (*P* < .02). However, mutational status of *IDH* was not assessed in 24% of tumors with central tumor location. An overview of histopathological diagnoses at primary surgery and molecular genetic status within each histopathological subtype is presented in Supplementary Table S2. Further details on applied methods for assessment of mutational status are available in Supplementary Table S3.

### Primary Surgery Including Timing of Treatment and Intraoperative Techniques

**Figure 3.**
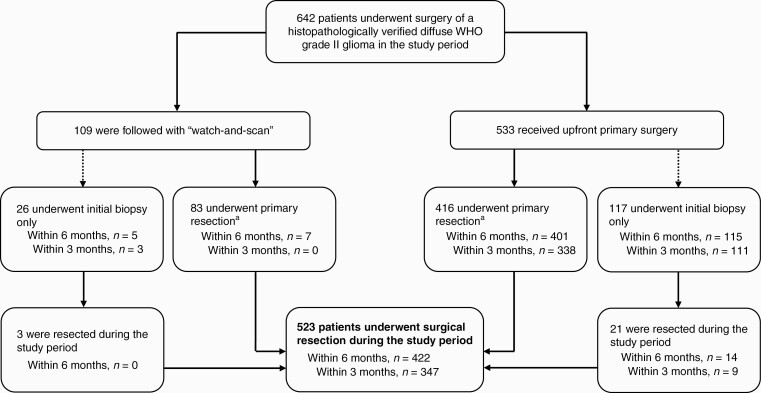
Flowchart displaying an overview over surgical strategies in the study period, and the proportion of patients who received surgical interventions within 3 and 6 months from radiological diagnosis. ^a^Time from radiological diagnosis to primary resection missing in 2 cases.

Surgical strategies are outlined in Figure 3. Further details on variations in pattern of primary surgical care including intraoperative imaging and techniques are available in Supplementary Table S4. Watch-and-scan was favored in 109 cases (17%) overall, ranging between 5% and 27% of cases across treating centers and with greater variability than expected when plotted against case volume with 1 significant outlier (Figure 2). There was no declining temporal trend during the 5-year period (*P* = 1.00). Watch-and-scan was not associated with tumor location or presumed eloquence but was advocated more frequently in asymptomatic patients with incidental DLGGs than in symptomatic patients, 44% (0%-64% across centers) vs 14% (0%-26%) of cases (*P* < .02). Further, patients in the watch-and-scan group had smaller tumors than patients who received upfront primary surgery, with median largest diameter measuring 30 (3-110) mm against 46 (10-110) mm, respectively (*P* < .02). Median duration of the watch-and-scan period calculated from the first MRI examination with DLGG suspect findings to primary surgery was 35.1 months (median 13.6-66.3 months across centers), and tumor growth was documented prior to surgery in 72% of patients during the watch-and-scan period.

**Figure 4.**
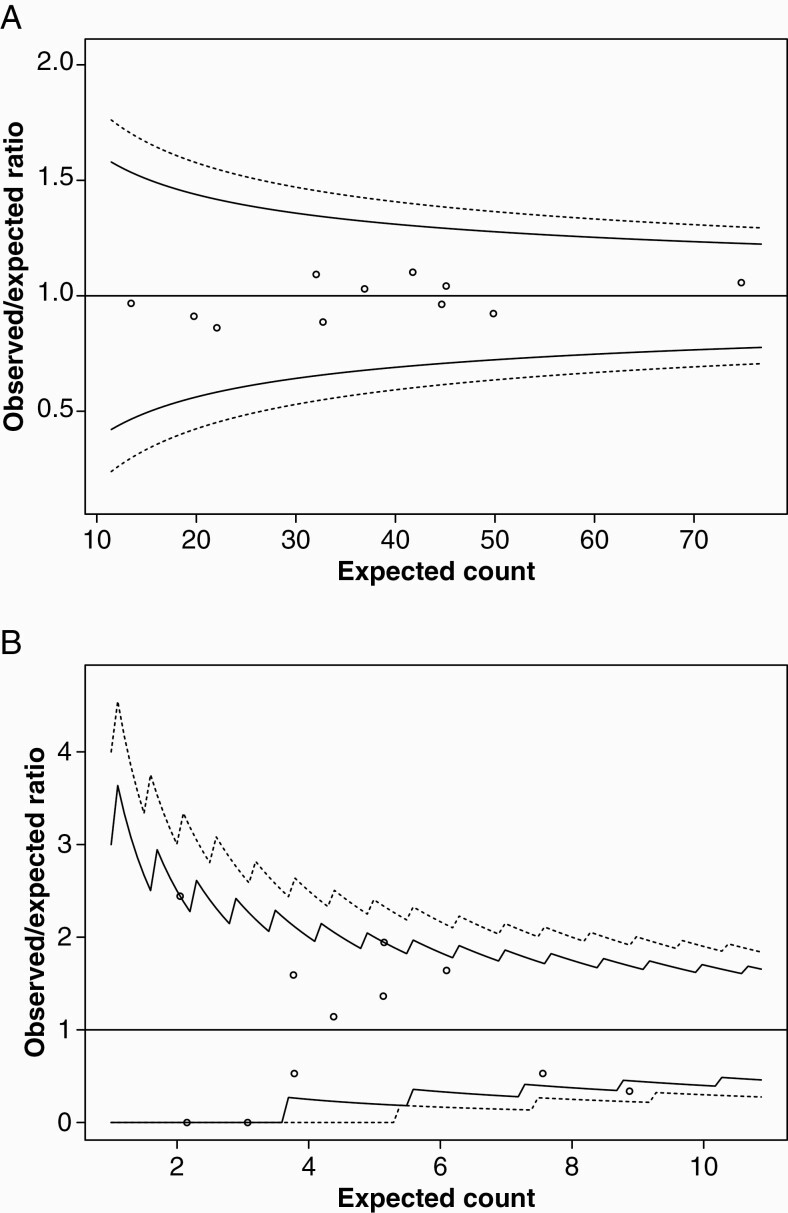
Funnel plots displaying inter-hospital variations in (A) resection rates within 6 months from radiological diagnosis adjusted for age in years, Karnofsky performance status score and year of surgery, (B) early postoperative radiotherapy plus PCV (procarbazine, lomustine, vincristine) adjusted for age in years, Karnofsky performance status score, year of surgery and primary surgical intervention (initial biopsy only vs primary resection).

Three hundred and forty-seven patients (54%, range 39%-67% across centers) were resected within 3 months from radiological diagnosis, and 422 patients (66%, 53%-73%) underwent resection within 6 months from radiological diagnosis. Elapsed time from radiological diagnosis to surgery was missing in 2 cases. For resection rates within 6 months, variability in expected range is displayed between centers when plotted against case volume (Figure 2) and expected variability with observed/expected ratios within 95% control limits is displayed when adjusted for age, KPS score, and year of surgery (Figure 4). In total, 523 patients (81%) were resected during the study period, ranging between 67% and 87% across treating centers. Surgical resection was more frequently advocated in younger patients, with median age of 41 (18-77) years, against 56 (19-82) years in the patient group who underwent biopsy only (*P* < .02). Moreover, resection rates were significantly lower in patients with centrally located tumors (30%, 0%-100% across centers) when compared to other tumor locations (85%, 67%-92%) (*P* < .02).

While neuronavigation based on preoperative MRI was extensively used at all treating centers (89%, range 81%-98% across centers), availability and choice of other tools and techniques for intraoperative guidance and surgical decision-making varied between centers (Supplementary Table S4). Intraoperative two-dimensional (2D) ultrasound (US) was the most widespread intraoperative imaging modality (47%, 9%-100%), whereas intraoperative MRI (iMRI) was available at 1 Norwegian and 1 Swedish center (toward the end of the study period) but used for intraoperative guidance during tumor resections in only 2 cases during the study period. Intraoperative brain mapping was performed in 157 out of 270 (58%, 0%-86%) primary resections in presumed eloquent locations, with an increasing time trend from 35% (0%-100%) to 74% (0%-100%) between 2012 and 2016 (*P* < .02). While awake surgery and mapping asleep were performed at all 6 Swedish centers, these techniques were utilized at 2 and 3 centers in Norway, respectively.

### Adjuvant Therapy, Including Timing of Treatment

Combined early adjuvant RT and chemotherapy (CHT) was administered in 154 patients (24%, range 13%-34% across centers), and this fraction was evenly distributed across histopathological subtypes and mutational status of *IDH* and 1p/19q. As seen in Figure 1, temozolomide (TMZ) was largely preferred alongside RT at some centers. The use of early RT + PCV was limited to 54 patients (8%, range 0%-20%), and variability between treating centers was more divergent than expected when adjusted for age, KPS score, year of surgery, and primary surgical intervention (biopsy vs resection), as shown in the funnel plot in Figure 4. However, there was an increasing temporal trend in the use of early RT + PCV during the study period, from 4% (0%-50%) in 2012 to 13% (0%-50%) in 2016 (*P* = .02). Further details on adjuvant therapy following primary surgery are available in Supplementary Table S5.

## Discussion

The objective of this multicenter study was to explore variations in DLGG management across all treating centers in 2 Scandinavian countries with similarly structured public health care systems. Surprisingly, no declining time trend was observed in watch-and-scan during the 5-year period, despite convincing evidence in favor of early resection.^1,8^ Further, in spite of an increasing time trend, the use of early adjuvant RT + PCV was limited to a small minority of patients, and with diverging practices between treating centers. However, landmark studies were published in the study period, and time to clinical implementation of newly acquired scientific evidence and subsequent practice changes may vary. Although differences in tumor classification and implementation of molecular markers are concerning, much of the variation can be attributed to the fact that patients were included in the transitional phase between the 2007 and 2016 WHO classification. The different use of techniques intending to spare functions and guiding surgical resection is likely to reflect the low evidence for most adjuncts and lack of well-conducted comparative studies. Altogether, many of the observed variations highlight major controversies associated with treatment of DLGGs and demonstrate that management of this heterogenous patient group still differs across treating centers, even in countries with universal coverage public health care where patients are treated free of charge, and where insurance policies do not influence the management. Some of the observed variations may presumably also reflect variability in clinical assessments and interpretation of the current evidence base and emphasize the need for high-level evidence to fill knowledge gaps.

Age-standardized incidence rates in the present study approximated 1/100 000 person-years which is comparable to incidence rates reported in the literature.^1–3^ Since the incidence of intracranial tumors is associated with the number of MRI scans carried out,^17^ local differences in the availability and use of MRI may influence DLGG incidence. Small fluctuations in diagnosis-specific incidence rates may also be caused by the often notoriously difficult diagnostic distinction between WHO grade II and III gliomas. Further, pronounced intra-tumoral heterogeneity implies a risk of tissue sampling bias. Consequently, under- and overgrading is not uncommon in studies exploring the concordance between histopathological diagnoses established from stereotactic biopsies compared to specimens obtained from open resections.^18^

The risk of erroneous glioma subtyping is reduced by incorporation of molecular markers that reflect genetic alterations occurring early and homogenously in tumorigenesis.^19^ However, patients were included in the transitional phase between the 2007 and 2016 WHO classification, and clinical implementation of *IDH* and 1p/19q in glioma diagnostics was widely varying across centers. The second cIMPACT-NOW (the Consortium to Inform Molecular and Practical Approaches to CNS Tumor Taxonomy-Not Official WHO) update from 2018 opened for refraining from direct 1p/19q testing in *IDH-mutant* DLGGs with unambiguous astrocytic phenotype, given strong p53 immunopositivity and/or definite loss of nuclear expression of ATRX (alpha-thalassemia/mental retardation, X-linked).^20^ Since the CRF solely contained questions regarding mutational status of *IDH* and 1p/19q, data on assessments of *ATRX* and *TP53* were not available.

Because subtle symptoms and deficits can be difficult to detect anamnestically and by standard neurological examination, there is a consensus that neuropsychological testing should ideally be an integral part of clinical preoperative work-up in DLGG patients.^21^ Yet, despite an increasing temporal trend, neuropsychological assessments were only performed to a small extent at most centers. In similarity with findings from an online survey on DLGG imaging practice among members of the European Low-Grade Glioma Network (ELGGN) with data from 24 European countries,^22^ there were pronounced variations in the application of advanced imaging techniques in preoperative work-up between treating centers, which may partly be attributed to the fact that most techniques are supported by limited evidence.^23^ Clinical utilization of amino acid PET may further be restricted by demanding logistics and high costs, because many of the included treating centers cannot produce their own tracers.

Surprisingly, watch-and-scan was pursued in 1 out of 6 patients in this cohort, with greater variability than expected across treating centers and 1 significant outlier. Further, there was no significant declining time trend, despite that studies supporting early resection were published in the study period.^1,8^ Because the progressive infiltrative nature of the disease and treatment-induced adverse effects both contribute to morbidity in DLGG patients, clinical decision-making is often challenging. The optimal treatment strategy in asymptomatic patients with small DLGG suspect lesions is debatable, especially in equivocal cases where benign lesions (eg, WHO grade I tumors) cannot be ruled out, when located in highly eloquent regions, or in older or very comorbid patients. Still, DLGGs always grow and there is a risk of malignant transformation at any time point. Thus, deferring treatment until demonstrated radiological growth or symptom onset is not a risk-free strategy. In a survey study within the ELGGN from 2015 including 21 European centers, 48% and 81% of respondents favored watch-and-scan as the preferred initial strategy in resectable and unresectable tumors, respectively, in all patients or depending on risk factors.^24^ However, most survey respondents stated an average duration of watch-and-scan of 6 months or less when advocated. By comparison, median duration of watch-and-scan ranged between 13.6 and 66.3 months across treating centers in the present cohort.

Surgical treatment was also awaited in some patients in the upfront primary resection group without any records of structured monitoring with MRI or cause of delay. In some cases, physicians may have chosen to await surgery due to comorbidity, without watch-and-scan being advocated as a deliberate strategy. Further, in ambiguous cases where other differential diagnoses were initially considered more likely, this may have caused delayed referrals and/or a decision to await surgery until supplementary diagnostic radiological work-up had been carried out or volumetric growth had been demonstrated.^25^

One-fifth were biopsied only in the study period, and resection rates were lower in older adults with centrally located tumors. As previously reported, *IDH-wildtype* gliomas had a predilection site for central brain regions and more frequently affected an older patient group than *IDH-mutant* gliomas.^26^ Aggressive biological behavior combined with an often more unfavorable tumor location is believed to partly explain the negative prognostic and predictive significance of age. Nevertheless, age-dependent variations in treatment strategies can also be caused by a certain element of therapeutic nihilism.^27,28^

Due to the risk of long-term toxicity, early adjuvant radiochemotherapy is usually reserved for patients with an unfavorable prognostic profile and presumed high risk of early malignant transformation. Results from the Radiation Therapy Oncology Group (RTOG) 9802 trial that demonstrated significantly prolonged OS in selected “high-risk” patients treated with combined adjuvant RT + PCV as compared to early RT alone were published in the study period.^9–11^ In the present cohort, the use of early RT + PCV was limited to a small minority of patients, albeit an increasing temporal trend was observed during the study period. Besides delay to clinical implementation, divergent practices between centers may also be partly attributed to variations in patient selection, since the definition of “high-risk” DLGG remains disputable^29^ and due to possible case-mix variations.

TMZ has some advantages in terms of administration method and toxicity profile, and in a survey study within the ELGGN from 2015, 76% of the included centers reported a preference for TMZ as first-line treatment over PCV.^24^ Correspondingly, TMZ was largely preferred over PCV at some centers in the present study. Because no trials to date have compared these chemotherapeutic regimens directly, the use of TMZ instead of PCV has less evidence base and remains controversial.^10,30,31^ An ongoing randomized phase III trial (NCT00887146) with estimated primary completion in 2025 is aiming to evaluate RT plus PCV vs RT with concomitant and adjuvant TMZ in patients with newly diagnosed 1p/19q co-deleted “high-risk” DLGGs and anaplastic gliomas.^32^

The retrospective study design and assessment of patient characteristics and clinical variables from non-standardized documentation in medical records are the main limitations of the study, and some data were thus incomplete or missing. Since the objective was to study variations in clinical practice, we refrained from attempting to homogenize data or imaging by performing central review. Three centers that accounted for 30% of the total case volume between 2012 and 2016 did not register data in 2017. Temporal trends and incidence rates were therefore calculated for 2012-2016. Despite existing guidelines, individual patient selection and treatment strategies will to some extent rely on subjective assessment. Besides, regional differences in organization and sub-specialization may contribute to variations in clinical management. Even though all treating centers advocate tissue diagnostics when a DLGG is radiologically suspected and this cohort for all practical purposes is an unselected population-based series, not all patients are fit enough for neurosurgical interventions at an acceptable risk/benefit ratio. Because the study exclusively includes patients who underwent surgery in the study period, a small number of patients may have been excluded due to inoperability. This selection effect is not random, as conservative treatment is more likely to be pursued in older comorbid patients with impaired functional status. Tumor growth was documented prior to surgery in the majority of patients followed by watch-and-scan. However, individual cases that may have undergone malignant transformation during the watch-and-scan period would thus no longer meet the inclusion criteria at the time of tissue diagnosis, and transformation rates may have been higher in patients harboring tumors with a more aggressive molecular biological profile. Consequently, the frequency of watch-and-scan may have been underestimated in this study. Moreover, the generalizability of findings to countries with differently structured health care systems is probably limited. However, one can speculate that the observed inter-hospital variations will be even greater in a setting without universal coverage public health care, where patient populations assumedly are more inhomogeneous and the resources more unevenly distributed.

## Conclusions

We describe the current pattern of care of DLGGs across treating centers in Norway and Sweden. Despite uniform public health care systems, geographical catchment regions that ensure population-based case selection, and existing national and international treatment guidelines, there were substantial differences in preoperative work-up, surgical strategies, and adjuvant treatment regimens across centers. Some of the observed disparities reflect controversies in DLGG management and highlight aspects where the knowledge base is deficient. Systematic registration of data can help improve negative outliers and enable future benchmark studies that evaluate progress over time and will make it possible to identify patterns associated with better or worse treatment results, including surgical resection grades, neurological outcomes, and survival. National or regional tumor boards might be a way to provide more homogenous and evidence-based care.

## Supplementary Material

npab054_suppl_Supplementary_Tables
