# Supplementary material for: Variations in the management of diffuse low-grade gliomas—A Scandinavian multicenter study
Source: Neurooncol Pract. 2021 Sep 4;8(6):706–17. doi: 10.1093/nop/npab054 (PMC8579093; doi:10.1093/nop/npab054)
Supplement: npab054_suppl_Supplementary_Tables [file npab054_suppl_supplementary_tables.docx]

| **Table S1.** List of study variables collected in the case report form. |
| --- |
| Patient characteristics |
| - Patient ID-number (specific for study) |
| - Center |
| - Year of surgery |
| - Inclusion date |
| - Year of birth - Sex |
| - Age at surgery |
| Baseline data |
| - Symptoms at (imaging) diagnosis |
| - - Seizure (yes/no) |
| - - Cognitive deficit (yes/no) |
| - - Motor deficit (yes/no) |
| - - Language deficit (yes/no) |
| - - Visual deficit (yes/no) |
| - - Headache / ICP related symptoms (yes/no) |
| - - Asymptomatic, MRI due to unrelated issues (yes/no) |
| - Clinical deterioration before surgery (yes/no) |
| - Karnofsky Performance Status score |
| - Neuropsychological assessment (yes/no), if yes |
| - - Neuropsychological impairment (yes/no) |
| Radiological data |
| Preoperative |
| - Date of MRI (or other imaging if MRI not performed) first demonstrating DLGG suspect lesion |
| - Initial “watch-and-scan” (yes/no) |
| - Demonstrated tumor growth prior to surgery (yes/no) |
| - MR spectroscopy (yes/no), if yes |
| - - Increased choline/NAA ratio (yes/no) |
| - Amino acid PET (yes/no), if yes |
| - - FET-PET |
| - - Methionine |
| - - Other (describe) |
| - Amino acid PET result (if yes above) |
| - - Focal hot spot |
| - - Homogenous increased uptake |
| - - Negative |
| - Main tumor location |
| - - Frontal |
| - - Temporal |
| - - Parietal |
| - - Occipital |
| - - Insula |
| - - Central/deep/basal ganglia |
| - Eloquence – as defined by Chang *et al.* in the UCSF score (yes/no) |
| - Multifocal (yes/no) |
| - Contrast enhancement (yes/no), if yes |
| - - Patchy/diffuse/weak |
| - - Nodular |
| - - Ring-like |
| - If yes above, contrast-enhancement developed in the period of ‘watch-and-scan’ (yes/no) |
| - Laterality |
| - - Left |
| - - Right |
| - - Bilateral/midline |
| - Largest diameter in millimeters |
| - Date of immediate preoperative MRI scan |
| Postoperative |
| - Postoperative MRI scan performed (yes/no) |
| - Significant DWI changes (yes/no/not assessed) |
| Tumor data |
| - WHO criteria used |
| - - 2007 |
| - - 2016 |
| - Histopathology according to WHO 2007 or 2016 classification |
| - - Astrocytoma |
| - - Oligodendroglioma |
| - - (Oligoastrocytoma) |
| - Molecular data, IDH status |
| - - Mutated |
| - - Wild-type |
| - - Not assessed |
| - Molecular data, 1p19q status |
| - - No codeletion |
| - - Codeletion |
| - - Not assessed |
| - IDH assessment |
| - - Immunohistochemistry (yes/no) |
| - - MLPA (yes/no) |
| - - Sequencing (yes/no) |
| - - Methylation (yes/no) |
| - - Other (yes/no) |
| - - Not assessed (yes/no) |
| - 1p19q assessment |
| - - FISH |
| - - MLPA |
| - - Sequencing |
| - - Methylation |
| - - Other |
| - - Not assessed |
| Standard of care description of primary surgery |
| - Date of surgery |
| - fMRI preoperatively (yes/no) |
| - DTI preoperatively (yes/no) |
| - Preoperative fMRI/DTI used intraoperatively (yes/no) |
| - nTMS preoperatively (yes/no) |
| - Neuronavigation (yes/no) |
| - Biopsy only (yes/no), if yes choose one |
| - - Frame-based stereotaxy |
| - - Frameless stereotaxy |
| - - Open biopsy |
| - - Other |
| - Awake surgery (yes/no) |
| - Mapping asleep (yes/no) |
| - If yes intraoperative brain mapping: |
| - Indication:   - - Language (yes/no) |
| - - - Motor (yes/no) |
| - - - Sensory (yes/no) |
| - - - Other parietal (yes/no) |
| - - - Visual (yes/no) |
| - - - Other (yes/no) |
| - - Biopolar (yes/no) |
| - - Monopolar (yes/no) |
| - - ECOG (yes/no) |
| - - Cortical stimulation (yes/no) |
| - - Subcortical stimulation (yes/no) |
| - - Relied on “negative mapping” (yes/no) |
| - - Positive mapping technique (yes/no) |
| - - Positive sites identified (yes/no) |
| - - Highest current used on direct stimulation |
| - - - [value] mA |
| - - - Missing |
| - - Continuous MEPs (yes/no), if yes |
| - - - Highest current used in continuous MEPs |
| - - - - [value] mA |
| - - - - Missing |
| - - Anesthesia in awake |
| - - - Asleep-awake-asleep |
| - - - Awake-awake-awake |
| - Intraoperative seizure (yes/no) |
| - Seizure prophylaxis (yes/no) |
| - - If yes, describe |
| - Stopped resection due to positive mapping (yes/no) |
| - Stopped resection due to perceived gross-total resection (yes/no) |
| - Laryngeal mask (yes/no) |
| - Spontaneous breathing (yes/no) |
| - Propofol for sedation (yes/no) |
| - Other drug for sedation, describe |
| - Scalp block (yes/no) |
| - 2D ultrasound (yes/no) |
| - 3D ultrasound (yes/no) |
| - Intraoperative CT (yes/no) |
| - Intraoperative MRI (yes/no) |
| - Microneurosurgery, i.e., use of microscope and microneurosurgical techniques (yes/no) |
| - Sonopet/CUSA (yes/no) |
| - Transcortical for access (yes/no) |
| - Transsulcal/open fissure for access (yes/no) |
| - Comment technique (describe) |
| - Elapsed time from preoperative MRI to surgery (in months) |
| - Elapsed time from DLGG suspect lesion on MRI to surgery (in months) |
| - Elapsed time from surgery to postoperative MRI (in months) |
| Other/later treatment |
| - Early radiotherapy – within 6 months of surgery (yes/no), if yes choose one |
| - - Proton |
| - - Photon |
| - Early chemotherapy – within 6 months of surgery or even neoadjuvant (yes/no), if yes choose one |
| - - PCV |
| - - Temozolomide |
| - - Lomustine |
| - - Other (describe) - Late radiotherapy – after 6 months following surgical treatment (yes/no), if yes choose one   - Proton   - Photon   - Gammaknife/stereotactic - Late chemotherapy – after 6 months following surgical treatment (yes/no), if yes choose one   - PCV   - Temozolomide   - Lomustine   - Other (describe) |
| CT: computed tomography; CUSA: cavitron ultrasonic surgical aspirator; DTI: diffusion tensor imaging; DWI: diffusion-weighted imaging; ECOG: electrocorticography; FISH: fluorescence in-situ hybridization; fMRI: functional magnetic resonance imaging; ICP: intracranial pressure; IDH: isocitrate dehydrogenase; MEP: motor evoked potential; MLPA: multiplex ligation-dependent probe amplification; MRI: magnetic resonance imaging; NAA: N-acetylaspartate; nTMS: navigated transcranial magnetic stimulation; PCV: procarbazine, vincristine, lomustine; PET: positron emission tomography; WHO: World Health Organization; UCSF: University of California, San Francisco. |

| **Table S2.** Histopathological diagnoses at primary surgery (1^st^ column), and molecular genetic status within histopathological subtypes based on available mutational assessments of isocitrate dehydrogenase (*IDH*) and 1p/19q. | | | | |
| --- | --- | --- | --- | --- |
|  | **Diagnosis according to the 2016 WHO Classification^a^** | **Total N**  **(%, range %)** | **Norway N**  **(%, range %)** | **Sweden N**  **(%, range %)** |
| **Astrocytoma** |  | **330 (51, 10-68)** | **136 (54, 42-68)** | **194 (50, 10-64)** |
| *IDH-mutant* |  | 159 (48, 4-79) | 78 (57, 41-65) | 81 (42, 4-79) |
| 1p/19q intact | **Astrocytoma, *IDH-mut*** | 84 (25, 0-67) | 35 (26, 16-38) | 49 (25, 0-67) |
| 1p/19q co-deletion | Oligodendroglioma | 1 (0, 0-2) | 0 (0) | 1 (1, 0-2) |
| 1p/19q not assessed | Inconclusive | 74 (22, 0-48) | 43 (32, 7-48) | 31 (16, 0-30) |
| *IDH-wildtype* | **Astrocytoma, *IDH-wt*** | 81 (25, 12-55) | 45 (33, 15-55) | 36 (19, 12-26) |
| *IDH* status not assessed | Inconclusive | 90 (27, 3-85) | 13 (10, 3-23) | 77 (40, 3-85) |
| **Oligodendroglioma** |  | **215 (33, 18-84)** | **75 (30, 19-42)** | **140 (36, 18-84)** |
| *IDH-mutant* |  | 118 (55, 17-100) | 57 (76, 54-100) | 61 (44, 17-100) |
| 1p/19q intact | Astrocytoma, *IDH-mut* | 4 (2, 0-13) | 1 (1, 0-5) | 3 (2, 0-13) |
| 1p/19q co-deletion | **Oligodendroglioma** | 112 (52, 15-100) | 56 (75, 54-100) | 56 (40, 15-88) |
| 1p/19q not assessed | Inconclusive | 2 (1, 0-12) | 0 (0) | 2 (1, 0-12) |
| *IDH-wildtype* | Astrocytoma, *IDH-wt* | 7 (3, 0-20) | 2 (3, 0-20) | 5 (4, 0-7) |
| *IDH* status not assessed | Inconclusive | 90 (42, 0-77) | 16 (21, 0-46) | 74 (53, 0-77) |
| **Oligoastrocytoma** |  | **97 (15, 2-25)** | **43 (17, 2-25)** | **54 (14, 6-24)** |
| *IDH-mutant* |  | 51 (53, 0-100) | 31 (72, 0-100) | 20 (37, 0-67) |
| 1p/19q intact | Astrocytoma, *IDH-mut* | 29 (30, 0-67) | 17 (40, 0-50) | 12 (22, 0-67) |
| 1p/19q co-deletion | Oligodendroglioma | 20 (21, 0-100) | 12 (28, 0-100) | 8 (15, 0-50) |
| 1p/19q not assessed | Inconclusive | 2 (2, 0-100) | 2 (5, 0-100) | 0 (0) |
| *IDH-wildtype* | Astrocytoma, *IDH-wt* | 7 (7, 0-33) | 5 (12, 0-20) | 2 (4, 0-33) |
| *IDH* status not assessed | Inconclusive | 39 (40, 0-100) | 7 (16, 0-100) | 32 (59, 0-100) |
| *IDH: isocitrate dehydrogenase; IDH-mut: IDH-mutant; IDH-wt: IDH-wildtype.*  ^a^Based on histopathological phenotype and available molecular markers (mutational status of *IDH* and 1p/19q). | | | | |

| **Table S3.** WHO classification and assessment of molecular genetic markers. | | | |
| --- | --- | --- | --- |
|  | **Total N**  **(%, range %)** | **Norway N**  **(%, range %)** | **Sweden N**  **(%, range %)** |
| **IDH assessment** | **423 (66, 24-98)** | **218 (86, 70-95)** | **205 (53, 24-98)** |
| Immunohistochemistry^a^ | 299 (71, 18-100) | 192 (88, 75-100) | 107 (52, 18-98) |
| MLPA | 35 (8, 0-69) | 17 (8, 0-25) | 18 (9, 0-69) |
| DNA sequencing | 88 (21, 0-82) | 22 (10, 4-14) | 66 (32, 0-82) |
| Methylation | 2 (0, 0-5) | 0 (0) | 2 (1, 0-5) |
| Other | 2 (0, 0-6) | 1 (0, 0-2) | 1 (0, 0-6) |
| Method not specified | 21 (5, 0-27) | 6 (3, 0-13) | 15 (7, 0-27) |
| Not assessed | 219 (34, 2-76) | 36 (14, 5-30) | 183 (47, 2-76) |
| **1p/19q assessment** | **412 (64, 42-79)** | **169 (67, 58-73)** | **243 (63, 42-79)** |
| FISH | 156 (38, 0-97) | 58 (34, 0-95) | 98 (40, 0-97) |
| MLPA | 184 (45, 0-100) | 93 (55, 0-100) | 91 (37, 0-100) |
| Sequencing | 2 (0, 0-9) | 2 (1, 0-9) | 0 (0) |
| Methylation | 6 (1, 0-12) | 0 (0) | 6 (2, 0-12) |
| Other | 12 (3, 0-15) | 8 (5, 0-15) | 4 (2, 0-7) |
| Method not specified | 52 (13, 0-67) | 8 (5, 0-10) | 44 (18, 0-67) |
| Not assessed | 230 (36, 21-58) | 85 (33, 27-42) | 145 (37, 21-58) |
| FISH: fluorescence in-situ hybridization; IDH: isocitrate dehydrogenase; MLPA: multiplex ligation-dependent probe amplification; WHO: World Health Organization.  ^a^Immunohistochemistry was supplemented with other assessment methods in 23 cases. | | | |

| **Table S4.** Standard of care description of primary surgery including intraoperative imaging and techniques. | | | |
| --- | --- | --- | --- |
|  | **Total N**  **(%, range %)** | **Norway N**  **(%, range %)** | **Sweden N**  **(%, range %)** |
| **Standard of primary surgical care** |  |  |  |
| Watch-and-scan | 109 (17, 5-27) | 55 (22, 5-27) | 54 (14, 10-19) |
| Initial biopsy only | 143 (22, 13-39) | 55 (22, 16-37) | 88 (23, 13-39) |
| Surgical resection |  |  |  |
| Within 3 months^a^ | 347 (54, 39-67) | 136 (54, 47-56) | 211 (54, 39-67) |
| Within 6 months^b^ | 422 (66, 53-73) | 156 (61, 60-68) | 266 (69, 53-73) |
| Missing | 2 (0, 0-3) | 1 (0, 0-3) | 1 (0, 0-2) |
| Time to primary surgery in months, median (lowest and highest median across centers)^c^ | 1.6 (0.8-2.3) | 1.4 (0.8-1.8) | 1.6 (1.1-2.3) |
| Upfront primary surgery | 1.2 (0.6-2.1) | 1.1 (0.6-1.4) | 1.2 (1.0-2.1) |
| Watch-and-scan | 35.1 (13.6-66.3) | 46.7 (18.7-66.3) | 22.5 (13.6-45.3) |
| **Intraoperative imaging and techniques for guiding resections** | 471 (94, 71-100) | 194 (97, 83-100) | 277 (92, 71-99) |
| Microneurosurgery |  |  |  |
| Missing | 1 (0, 0-3) | 0 (0) | 1 (0, 0-3) |
| Ultrasonic surgical aspirator | 357 (72, 25-100) | 169 (85, 25-98) | 188 (63, 43-100) |
| Missing | 1 (0, 0-3) | 0 (0) | 1 (0, 0-3) |
| Neuronavigation | 445 (89, 81-98) | 182 (91, 90-96) | 263 (88, 81-98) |
| Missing | 1 (0, 0-3) | 0 (0) | 1 (0, 0-3) |
| Preoperative fMRI/DTI used intra-operatively^d^ | 89 (45, 0-100) | 25 (35, 0-83) | 64 (52, 0-100) |
| Missing | 57 (29, 0-100) | 21 (29, 0-100) | 36 (29, 0-100) |
| 2D ultrasound | 234 (47, 9-100) | 75 (38, 13-100) | 159 (53, 9-96) |
| Missing | 1 (0, 0-3) | 0 (0) | 1 (0, 0-3) |
| 3D ultrasound | 40 (8, 0-98) | 40 (20, 0-98) | 0 (0) |
| Missing | 1 (0, 0-3) | 0 (0) | 1 (0, 0-3) |
| Intraoperative CT | 0 (0) | 0 (0) | 0 (0) |
| Intraoperative MRI | 2 (0, 0-3) | 2 (1, 0-3) | 0 (0) |
| Awake surgery^e^ | 96 (36, 0-79) | 41 (44, 0-79) | 55 (31, 14-41) |
| Mapping asleep^e^ | 108 (22, 0-45) | 20 (10, 0-38) | 88 (29, 19-45) |
| 2D: two-dimensional; 3D: three-dimensional; CT: computed tomography; DLGG: diffuse low-grade glioma; DTI: diffusion tensor imaging; fMRI: functional magnetic resonance imaging; MRI: magnetic resonance imaging.  ^a^Surgical resection within 3 months from when a DLGG was radiologically suspected.  ^b^Surgical resection within 6 months from when a DLGG was radiologically suspected. Including the 7 patients in the “watch-and-scan” group who were resected within 6 months from radiological diagnosis and the 11 patients who underwent initial biopsy only and resection within 6 months from radiological diagnosis.  ^c^Elapsed time from the first MRI examination with DLGG suspect findings to primary surgery. Time from radiological diagnosis to surgery missing in 2 cases.  ^d^Calculated from the proportion of cases with presumed eloquent location where preoperative fMRI and/or DTI had been carried out for noninvasive functional mapping.  ^e^Within cases located in presumed eloquent locations. | | | |

| **Table S5.** Adjuvant therapy following primary surgery. | | | |
| --- | --- | --- | --- |
|  | **Total N**  **(%, range %)** | **Norway N**  **(%, range %)** | **Sweden N**  **(%, range %)** |
| Early radiotherapy^a^  If yes: | **250 (39, 16-57)** | **78 (31, 16-43)** | **172 (44, 25-57)** |
| Photon | 198 (79, 56-100) | 72 (92, 56-100) | 126 (73, 60-93) |
| Proton | 40 (16, 0-34) | 1 (1, 0-5) | 39 (23, 7-34) |
| Missing | 12 (5, 0-44) | 5 (6, 0-44) | 7 (4, 0-30) |
| Missing | 1 (0, 0-2) | 0 (0) | 1 (0, 0-2) |
| Late radiotherapy^b^  If yes: | **153 (24, 10-34)** | **69 (27, 16-34)** | **84 (22, 10-30)** |
| Photon | 101 (66, 14-100) | 63 (91, 60-100) | 38 (45, 14-73) |
| Proton | 32 (21, 0-86) | 0 (0) | 32 (38, 7-86) |
| Gammaknife/stereotactic | 10 (7, 0-23) | 1 (1, 0-7) | 9 (11, 0-23) |
| Missing | 10 (7, 0-40) | 5 (7, 0-40) | 5 (6, 0-25) |
| Missing | 1 (0, 0-2) | 0 (0) | 1 (0, 0-2) |
| Early chemotherapy^c^ | **147 (23, 6-47)** | **52 (20, 11-27)** | **95 (24, 6-47)** |
| If yes: |  |  |  |
| Lomustine | 15 (10, 0-58) | 0 (0) | 15 (16, 0-58) |
| PCV | 57 (39, 0-75) | 26 (50, 0-75) | 31 (33, 0-74) |
| Temozolomide | 68 (46, 25-100) | 20 (38, 25-100) | 48 (51, 26-100) |
| Other | 4 (3,0-20) | 4 (8, 0-20) | 0 (0) |
| Missing | 3 (2,0-33) | 2 (4, 0-33) | 1 (1, 0-3) |
| Missing | 1 (0, 0-2) | 0 (0) | 1 (0, 0-2) |
| Late chemotherapy^d^ | **196 (31, 10-47)** | **74 (29, 10-35)** | **122 (31, 12-47)** |
| If yes: |  |  |  |
| Lomustine | 27 (14, 0-48) | 0 (0) | 27 (22, 0-48) |
| PCV | 51 (26, 4-67) | 26 (35, 24-67) | 25 (20, 4-62) |
| Temozolomide | 105 (54, 23-75) | 42 (57, 33-75) | 63 (52, 23-71) |
| Other | 12 (6, 0-22) | 6 (8, 0-16) | 6 (5, 0-22) |
| Missing | 1 (1, 0-8) | 0 (0) | 1 (1, 0-8) |
| Missing | 1 (0, 0-2) | 0 (0) | 1 (0, 0-2) |
| Early radiochemotherapy | **154 (24, 13-34)** | **50 (20, 13-26)** | **104 (27, 14-34)** |
| Early RT+PCV | 54 (35, 0-86) | 29 (58, 0-86) | 25 (24, 0-77) |
| Early RT+TMZ | 73 (47, 14-100) | 17 (34, 14-100) | 56 (54, 15-100) |
| Missing | 1 (0, 0-2) | 0 (0) | 1 (0, 0-2) |
| Surgical resection within 6 months followed by early radiochemotherapy | **97 (15, 0-25)** | **26 (10, 0-16)** | **71 (18, 0-25)** |
| Early resection followed by early RT+PCV | 29 (30, 0-100) | 14 (54, 38-100) | 15 (21, 0-70) |
| Early resection followed by early RT+TMZ | 45 (46, 0-76) | 9 (35, 0-54) | 36 (51, 20-76) |
| CHT: chemotherapy; PCV: procarbazine, lomustine and vincristine; RT: radiotherapy; TMZ: temozolomide.  ^a^Initiation of radiotherapy within 6 months postoperatively.  ^b^Initiation of radiotherapy > 6 months postoperatively.  ^c^Neoadjuvant or within 6 months postoperatively.  ^d^Initiation of chemotherapy > 6 months postoperatively. | | | |
